# Taekwondo exercises for women improve quality of life, physical self-defence skills, and psychological resilience

**DOI:** 10.3389/fpsyg.2025.1638975

**Published:** 2025-07-30

**Authors:** Ahmet Yıkılmaz, Coşkun Yılmaz, Atıf Çiçekli, Önder Eryılmaz, Cemalettin Budak, Musa Uyar, Tülay Ceylan, Özkan Güler, Fatma Neşe Şahin

**Affiliations:** ^1^Faculty of Sport Sciences, Iğdır University, Igdır, Türkiye; ^2^Kelkit Aydın Doğan VC, Gümüşhane University, Gümüşhane, Türkiye; ^3^Governorship of Amasya, Amasya, Türkiye; ^4^Faculty of Education, Amasya University, Amasya, Türkiye; ^5^Faculty of Sport Sciences, Erzincan Binali Yıldırım University, Erzincan, Türkiye; ^6^Department of Physical Education and Sports, National Defense University, Ankara, Türkiye; ^7^Postgraduate Education Institute, Ondokuz Mayıs University, Samsun, Türkiye; ^8^Faculty of Sport Sciences, Ankara University, Ankara, Türkiye

**Keywords:** combat sports, mental health, Taekwondo, physical self-defense, psychological resilience, quality of life, sport psychology

## Abstract

**Background:**

The extant research on Taekwondo has focused primarily on the physiological effects of training, with limited interest in psychological resilience, self-defence and quality of life levels. The present study examined the effects of Taekwondo exercises on psychological resilience, self-defence and quality of life levels in healthy female subjects.

**Methods:**

The present study comprised 30 healthy, sedentary female subjects. The subjects were randomly divided into two groups: one group participated in Taekwondo training (TG), while the other group served as the control group (CG). The sample sizes for both the TG and CG groups were 15. The TT group underwent conventional Taekwondo instruction, while the CG group maintained their habitual routine and refrained from sporting pursuits. The Brief Psychological Resilience Scale, the Physical Self-Defence Scale for Women, and the SF-12 Quality of Life Scale were administered before and after the six-week training period.

**Results:**

The 6-week Taekwondo training programme led to significant improvements in both physical health (PH) (*p* = 0.049) and mental health (MH) (*p* < 0.01) scores in female participants. It did not produce a significant change in psychological resilience (PR) scores (*p* > 0.05). It produced significant improvements in self-defence against simple physical attacks (SP) (*p* = 0.004) and self-defence against dangerous physical attacks (DP) (*p* = 0.041) scores.

**Conclusion:**

The positive effects of Taekwondo training on psychological resilience, physical self-defence and quality of life levels have been demonstrated in healthy female subjects. This study provides valuable insights into the impact of Taekwondo exercises on psychological resilience, physical self-defence and quality of life levels. The findings of this study can guide future intervention and programme design in the context of sports psychology.

## 1 Introduction

In the contemporary era, the prospect of leading a healthy life is no longer confined to enhancing physical fitness levels; individuals can also achieve this by receiving support in psychological, social, and cognitive domains. In this context, scientific studies have demonstrated that regular physical activity exerts multifaceted positive effects on both physiological and psychological health indicators (Yılmaz et al., 2021; Stanković et al., 2022; Ciaccioni et al., 2024; Çelikel et al., 2025; Qiu et al., 2025). The impact of physical activity on mental health components, such as stress management, psychological resilience, and self-efficacy, contributes to the sustainable enhancement of an individual’s quality of life.

Martial arts, particularly those with structured training systems such as Taekwondo, have been shown to engender improvements in physical fitness (Toskovic, 2001; Fong and Ng, 2011; Roh et al., 2018). In addition to this, they have also been demonstrated to foster significant benefits in terms of ethical values, self-discipline, emotional control and social harmony (Roh et al., 2018). The pedagogical application of Taekwondo has been demonstrated to encourage the development of multifaceted personality traits in participants, including self-control, respect, responsibility, leadership, and self-confidence (Kim et al., 2021). Consequently, Taekwondo is regarded not solely as a martial art for both children and adults, but also as a value-based educational instrument that fosters character development (Toskovic, 2001; Epstein and Krasner, 2013).

From a feminist standpoint, the participation of women in physical activity has been shown to enhance health indicators and cultivate resilience against stress factors stemming from gender roles (White et al., 2005; Obi et al., 2023). Taekwondo has been demonstrated to enhance women’s physical self-defence capabilities, thereby reinforcing their sense of self-efficacy and strengthening their psychological resilience (Yılmaz et al., 2024). Psychological resilience is defined as an individual’s ability to respond adaptively to stress, trauma, and adverse life events (Rutter, 2000; Luthar et al., 2003; Garrido-Muñoz et al., 2024). This capacity is influenced by various factors, including self-control, social support, and cognitive flexibility. It has been documented that individuals who possess high psychological resilience are able to develop more effective coping strategies in the face of challenging life conditions and are able to sustain their quality of life (Epstein and Krasner, 2013).

Quality of life, on the other hand, is a subjective assessment that encompasses a multidimensional structure including physical health, psychological state, social relationships, and environmental factors (Ciaccioni et al., 2022; Frisch, 2024). However, in the literature, quality of life is mostly addressed from a psychological health perspective; sociological determinants such as gender, socioeconomic status, educational level, and living environment (urban/rural) are not sufficiently considered. This situation highlights the need for a more comprehensive approach to evaluating interventions specific to women’s quality of life (Nutakor et al., 2023).

Recent studies have demonstrated the efficacy of Taekwondo training in enhancing physical fitness and body composition in women. In addition, it has been shown to be an effective tool in the management of psychological issues such as depression, anxiety, and social isolation (Gao et al., 2025). Moreover, given its inherent capacity to foster cross-cultural social interactions, Taekwondo has been demonstrated to enhance social integration and cultural awareness levels, particularly among university-aged women (de Oliveira et al., 2023). However, studies examining the effects of martial arts on women are mostly cross-sectional in design and insufficient in explaining cause-and-effect relationships (Kim and Nam, 2021; Kim et al., 2021; Davies and Deckert, 2020; Croom, 2022; Gao et al., 2025). This underscores the necessity for longitudinal and intervention-based research methodologies. Moreover, while the extant literature predominantly focuses on martial arts, there is a paucity of systematic studies specific to Taekwondo. The objective of this study is to analyse the effects of a six-week Taekwondo training programme on quality of life, psychological resilience, and physical self-defence levels in female university students. The primary hypothesis of the study is that structured six-week Taekwondo exercises will enhance quality of life, psychological resilience, and physical self-defence levels. The findings of this study have the potential to inform the development of intervention programmes that address the physical and psychosocial health needs of female university students.

## 2 Materials and methods

### 2.1 Participants

Thirty healthy female subjects were included in the study. The study was designed as a randomised, controlled experimental study. Participants were randomly assigned to two different groups: the Taekwondo group (TG) and the Control (CG) group. The GPower 3.1 programme was used to determine the required number of participants. The results of the power analysis of the sample study showed that the study could be completed with 12 subjects in each group (effect size: 0.80; actual power: 0.89). To account for possible losses, 15 subjects were allocated to each group (Control and Taekwondo), adding 25% to the total of 30 subjects. To determine which group the participants in the sample would be assigned to, numbers between 1 and 30 were randomly assigned to two groups using a computer program.^1^ Participants with chronic or any other illnesses were excluded from the study. Informed consent was obtained from all participants verbally and in writing before the study began.

### 2.2 Experimental design

**Figure 1:**
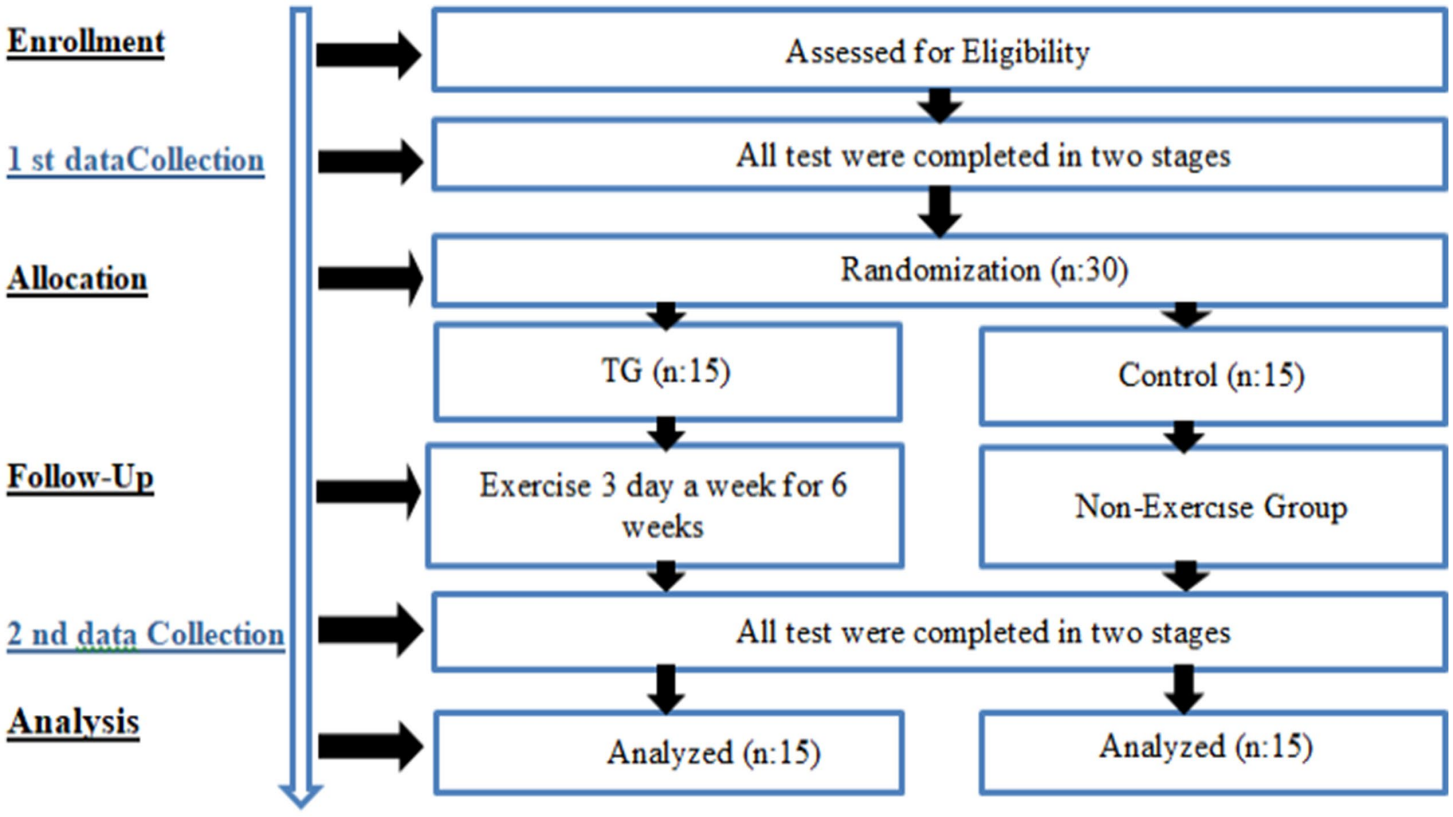
Experimental design.

Healthy female participants in the study were asked to visit the laboratory three times. During the first visit, they were given information about the training procedures and scales. Each subject was given a detailed explanation of the Taekwondo training procedure. During the next visit, which took place 1 week later, pre-exercise measurements were taken and the values were recorded. At the end of the six-week training programme, final measurements were taken during the third and final visits (see Figure 1).

### 2.3 Body composition measurement

In the study, body composition was measured with Gaia 359 Plus Body-pass bioelectrical impedance analyser to indicate that the participants had similar body characteristics. The Gaia 359 Plus BodyPass was used to determine the subject’s height and body weight (see Table 1).

**Table 1:** Descriptives.

Group	Height		Weight		Age	
	*X*	S.D.	*X*	S.D.	*X*	S.D.
Taekwondo	160.20	5.13	57.33	9.19	20.67	1.11
Control	161.93	4.62	58.40	5.25	20.80	1.15

### 2.4 Physical self-defense scale for women

The scale developed by Uyar (2022) comprises 12 items and two sub-dimensions: self-defense against dangerous physical attacks (DP) and self-defense against simple physical attacks (SP). The scale does not include any items that can be reverse scored. The lowest possible score that can be attained on this five-point Likert-type scale, comprising two sub-dimensions and 12 items, is 12, while the highest score is 60. The initial sub-dimension, encompassing self-defense against dangerous physical attacks, comprises six items. A high score within this dimension indicates that women possess elevated levels of self-defense against physical attacks that they perceive as more dangerous. The remaining sub-dimension assesses women’s capacity for self-defense in the context of physical attacks that they perceive as less dangerous, comprising six items. A high score in this sub-dimension indicates that women possess a robust capacity for self-defense against physical attacks that they perceive as less dangerous. In this study, the alpha coefficients were 0.894 for the dangerous assault sub-dimension and 0.807 for the simple assault sub-dimension. It was determined that the scale had very high reliability results such as 0.80 ≤ *α* < 1.00.

### 2.5 The Quality of Life Scale (QOLS)

The Short Form-36 (SF-36) was developed by Ware et al. (1995) as a means of measuring quality of life. The SF-12 Quality of Life Scale was constructed by selecting 12 items from eight subheadings of the SF-36. A Turkish validity and reliability study was conducted by Soylu and Kütük (2021). The scale enquiry concerns the individual’s functional status, well-being and general health perception. The responses to questions pertaining to physical and emotional status are dichotomous (yes/no), whereas other questions employ a Likert-type scale with options ranging from 3 to 6. The Mental Component Summary (MCS) score is derived from the Mental Health, Emotional Role, Social Functioning, and Energy subcategories, while the Physical Component Summary (PCS) score is calculated from the Physical Role, Physical Functioning, General Health, and Body Pain subcategories. Scoring is on a scale of 0 to 100. A higher score is indicative of superior health status. The Cronbach’s alpha coefficient of the SF-12 Short Health Scale was calculated as *α*: 0.73 for the physical subscale and *α*: 0.72 for the mental subscale (Soylu and Kütük, 2021). The alpha coefficient of these two sub-dimensions, which explained 57.48% of the total variance, was calculated as 0.555 for the physical subscale and 0.698 for the mental subscale. The SF-12 scale was scored on the OrthoToolKit website.

### 2.6 The Brief Psychological Resilience Scale (SPRS)

The Brief Psychological Resilience Scale (SPRS) was developed by Smith et al. (2008) and adapted to Turkish culture by Doğan (2015). The scale under consideration was developed for the purpose of measuring individuals’ levels of psychological resilience. It consists of six items and a single sub-dimension on a 5-point Likert scale. The scale incorporates reverse-coded items (2, 4, 6). Following the correction of reverse-coded items, the highest attainable score on the scale is 30, whereas the lowest is 6. A high score on the scale is indicative of a high level of psychological resilience. The scale is characterised by ease of use and scoring, practicality, and the capacity to be administered in a relatively brief period. Doğan (2015) determined the Cronbach’s alpha reliability of the scale to be 0.81 and the internal consistency coefficient to be 0.83. The findings of this study indicate that the scale is a highly valid and reliable measurement tool that can be applied in Turkish culture. In the present study, the Cronbach’s alpha reliability coefficient of the scale was determined to be 0.794.

### 2.7 Weekly training program

The training programme involves participants performing Taekwondo kyorugi (basic and combined strikes) exercises for a duration of 60 min per day, on 3 days per week, over a period of 6 weeks. The extant literature indicates that a period of between 6 and 12 weeks, with a minimum of 3 days per week, is a very important period for development (Hudelmaier et al., 2010). Consequently, a six-week period was deemed to be an appropriate duration for the study. The exercise programme was meticulously structured in three phases, informed by the insights of three Taekwondo experts. The initial phase of the training regime was a five-minute running routine of low intensity, followed by a ten-minute series of exercises designed to improve agility and coordination. These comprised two steps, side steps, cross steps, two-foot jumps, single-leg jumps, squat jumps, high knees (×10), single knee, cutting kicks, one step back and two steps back. A stretching phase consisting of 5 min of dynamic stretching was performed with the objective of preventing muscle stiffness and facilitating relaxation of the body. The fundamental phase was administered to subjects for a duration of 60 min on 3 days of the week (Tuesday, Thursday, and Saturday) (Yılmaz et al., 2024) (see Table 2).

**Table 2:** Six week programme.

Week	Implemented program
1 week	Apchagi, Dolryeo chagi, Naryeo chagi, (each set of movements was repeated 3 times for 4 min. Rest was given for 1 min between each set).
2 week	Ggeulousi apchagi, Apbal dolryeochagi, (each set of movements was repeated 3 times for 4 min. Rest was given for 1 min between each set).
3 week	Jump neryeo jjikgi, Neryeo jjikgi, (each set of movements was repeated 3 times for 4 min. Rest was given for 1 min between each set).
4 week	Dolgae chagi, Naraechagi, Punch, Dwitchagi, Dwihooryeo chagi, (each set of movements was repeated 3 times for 4 min. Rest was given for 1 min between each set).
5 week	Combined stroke training (each set of combined movements was repeated 3 times for 4 min. 1 min rest was given between each set).
6 week	Combined stroke training (each set of combined movements was repeated 3 times for 4 min. 1 min rest was given between each set).

### 2.8 Statistical analysis

Statistical analyses were performed via SPSS (Version 21.0 for Windows, Chicago, IL, United States) software, with the statistical significance set at 0.05. The Shapiro–Wilk normality test was performed to determine the homogeneity of the sample. Each pre-test and post-test differences were determined by paired comparison test (paired *t*-test), and inter-group differences were determined by one-way analysis of variance with post-test and pre-test difference values. In addition, in the comparison of paired groups, the effect size was calculated according to Cohen’s *d* formula ((M2 − M1)/SD pooled). Moreover, it was interpreted as follows: 0–0.19 insignificant, 0.20–0.59 small, 0.6–1.19 moderate, 1.20–1.99 large, and ≥2.00 very large (see Table 3).

**Table 3:** Mean, reliability, skewness and kurtosis values of the scales.

	Sub-dimensions	Number of items	*X*	S.D.	*α*	Kurtosis	Skewness
Short Psychological Resilience Scale		6	18.93	3.72	0.693	0.565	1.043
The quality of life scale	Physical subscale	6	44.40	8.5	0.723	−0.765	−0.469
	Mental subscale	6	44.33	9.76	0.744	−0.724	−0.215
Physical self-defense scale for women (*α*: 0.933)	Dangerous physical	6	16.31	5.96	0.819	−0.365	0.800
	Simple physical	6	20.18	4.79	0.918	−0.267	0.240

*α*, Cronbach’s alpha; X, mean; S.D., standard deviation.

## 3 Results

**Figure 2:**
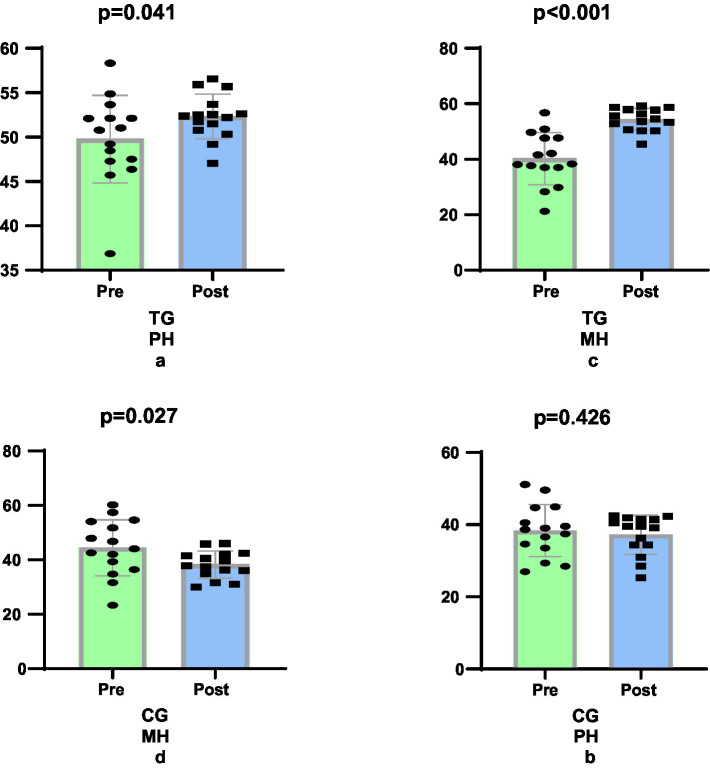
Comparison quality of life levels pre-post training.

The study revealed that the 6-week Taekwondo training programme resulted in an 8.11% greater improvement in PH scores in women in the TG (e.s. 0.654, 5.14%, *p* = 0.041) group compared to the CG (e.s. 0.179, −2.97%, *p* = 0.426) group (*p* = 0.049, Figures 2a,b). In terms of MG scores, the TG (e.s. 1.956, 35.04%, *p* < 0.001) group showed a 49.03% greater improvement in MH scores compared to the CG (e.s. 0.766, 13.99%, *p* = 0.027) group (*p* < 0.001, Figures 2c,d).

**Figure 3:**
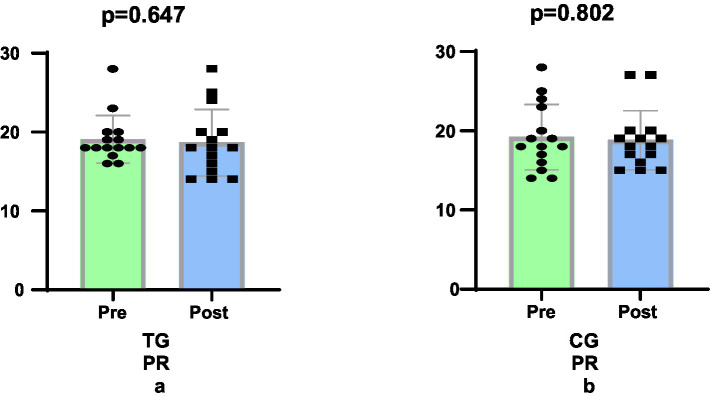
Comparison of psychological resilience levels pre-post training.

The study found that as a result of the 6-week Taekwondo training programme, TG (e.s. 0.109, 2.09%, *p* = 0.647) group showed a similar improvement in PR scores compared to the CG (e.s. 0.101, −2.08%, *p* = 802) group (*p* = 1.000, Figures 3a,b).

**Figure 4:**
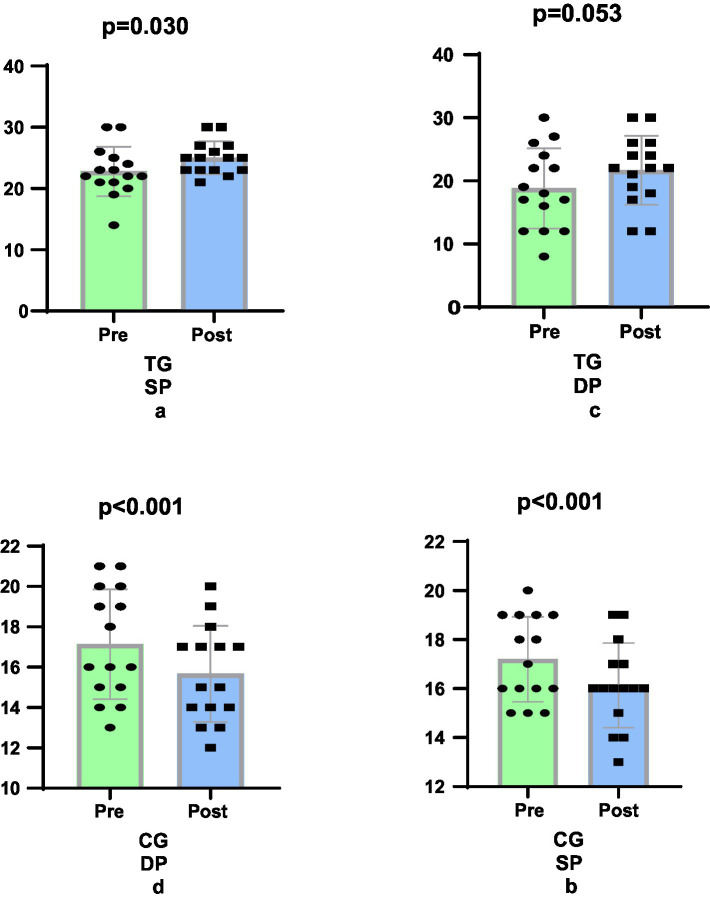
Comparison of physical self-defense levels pre-post training.

The study revealed that the 6-week Taekwondo training programme resulted in a 17.82% greater improvement in SP scores in women in the TG (e.s. 0.637, 11.60%, *p* = 0.030) group compared to the CG (e.s. 0.617, −6.22%, *p* < 0.001) group (*p* = 0.004, Figures 4a,b). In terms of DP scores, the TG (e.s. 0.485, 20.38%, *p* = 0.053) group showed a 28.9% more significant improvement in DP scores compared to the CG (e.s. 0.571, 8.52%, *p* < 0.001) group (*p* = 0.041, Figures 4c,d).

## 4 Discussion

The primary objective of this study is to examine the effects of 6 weeks of Taekwondo training on psychological resilience, self-defence and quality of life levels in healthy female subjects. The primary conclusions of this study suggest that a six-week Taekwondo training programme administered to healthy women enhances participants’ quality of life, self-defence skills, and psychological resilience levels. The central hypothesis of this study, which posits that a structured 6-week Taekwondo exercise programme improves quality of life, psychological resilience, and physical self-defence levels, was confirmed.

Taekwondo (TKD) is an Olympic sport that involves the disciplined application of different movement forms through the harmonious synchronisation of a peaceful mind and body. These unique characteristics enable balanced personal development and become a way of life (USATKD, 2022). Unlike other martial arts, participating in and practising Taekwondo strength, endurance, flexibility, coordination, concentration, goal setting, decision making, self-defence, self-respect, control, quality of life, discipline, and other physical, physiological, psychological, and life skills advantages (Yılmaz et al., 2024; Nair, 2020; Burke et al., 2007). Therefore, it can be said that Taekwondo develops the individual cognitively, physically, emotionally, and socially (Lakes et al., 2013).

The six-week Taekwondo training program resulted in substantial enhancements in both physical health (PH) and mental health (MH) scores among the female participants. In terms of physical health (PH): The Taekwondo group (TG) demonstrated a 8.11% greater improvement in comparison to the control group (CG) (*p* = 0.049). With regard to mental health (MH): A 49.03% greater improvement was observed in the TG group compared to the CG (*p* < 0.001). The findings underscore the efficacy of Taekwondo training in enhancing the physical and mental well-being of women, thereby underscoring its significant contribution to mental health. These findings are consistent with those reported in previous studies.

A multitude of studies have been conducted to examine the impact of Taekwondo training on quality of life. These studies have revealed that this sport has a positive effect on physical, psychological, and social areas. Cromwell et al. (2007) underscored the multifaceted nature of Taekwondo, asserting that it functions not only as a physical exercise but also as a catalyst for enhancing psychological factors, including discipline, patience, and mental endurance. This comprehensive approach, they contend, leads to a marked improvement in overall quality of life. As demonstrated in the study by Yılmaz et al. (2024), a six-week Taekwondo training program yielded positive outcomes, including enhanced self-control, improved quality of life, and augmented self-defense skills, in sedentary women. Lakes and Hoyt’s (2004) study on the psychosocial effects of Taekwondo on children reported significant improvements in skills such as self-confidence, self-discipline, and stress management. The study also found a positive contribution to children’s quality of life. Kim et al. (2015) study, the impact of Taekwondo training on the physical health and quality of life of female university students was examined. The findings indicated that regular Taekwondo training led to improvements in overall quality of life, with significant increases observed in muscle strength, flexibility, and cardiovascular health. In their study, Fong and Ng (2011) examined the effects of Taekwondo on elderly individuals. Their findings indicated that Taekwondo improved balance, flexibility, and overall quality of life. Additionally, it enhanced psychosocial factors such as social participation and self-esteem. In their research on martial arts such as Taekwondo, Burke et al. (2007) reported that in addition to supporting physical health, these sports also have significant effects on mental health and quality of life, such as reducing stress, increasing self-expression and self-control. According to the findings of Jung et al. (2017), regular Taekwondo training has been demonstrated to enhance quality of life by increasing physical health parameters, including muscle strength, flexibility, and cardiovascular endurance. Gao et al. (2025) posit that Taekwondo is an effective method of alleviating depression, particularly in older women. Ziaee et al. (2012) demonstrated in their study on young men that regular Taekwondo training improved psychological well-being and overall quality of life by reducing depression, anxiety, and stress levels. Kim et al. (2012) discovered that a Taekwondo exercise program has the potential to enhance physical fitness, physical function, and quality of life in women diagnosed with osteoarthritis. Chyu et al. (2013) posited that martial arts training may be a viable and efficacious approach to enhance body composition and quality of life in overweight/obese premenopausal women. Kim et al. (2014) posited that Taekwondo training exerts a positive effect on social well-being, thereby enhancing social skills, teamwork, and social participation. These social interactions, in turn, have been demonstrated to improve quality of life. Douris et al. (2015) reported that regular Taekwondo training in middle-aged individuals increased physical fitness, strengthened resilience against stress, and provided positive effects on quality of life. The majority of these studies have concluded that Taekwondo contributes to an enhanced overall quality of life by improving not only physical but also psychological and social well-being. However, further research is necessary, particularly on specific populations such as women.

With respect to psychological resilience (PP) scores, no statistically significant differences were observed between the Taekwondo and control groups. The findings of this study demonstrate that Taekwondo training exerts minimal influence on psychological resilience and does not result in an augmentation of psychological resilience in the short term. This finding suggests that studies such as Toskovic (2001) and Roh et al. (2018) may attribute the positive effects on psychological resilience to long-term or more intensive training protocols. Furthermore, the development of a complex and multicomponent structure, such as psychological resilience, may necessitate prolonged interventions and studies that consider individual differences (Selucik and Dilek, 2020; Moore et al., 2020). A review of the extant literature indicates that Taekwondo training fosters the development of numerous components of psychological resilience, including self-control, stress management, self-confidence, social bonds, and mental resilience (Selucik and Dilek, 2020; Moore et al., 2020; Douris et al., 2015; Kim et al., 2014; Lakes et al., 2013). The following text is intended to provide a comprehensive overview of the subject matter. Consequently, Taekwondo training can be regarded as a protective and developmental intervention tool, particularly for vulnerable groups such as children, youth, and women.

The 6-week Taekwondo training program resulted in substantial enhancements in SP and DP scores among the female participants. In consideration of the SP: Taekwondo group (TG), a 17.82% increase in improvement was demonstrated in comparison to the control group (CG) (*p* = 0.004). With regard to the DP, the TG group exhibited a 28.9% greater improvement in comparison to the CG (*p* = 0.041). These findings suggest that Taekwondo training is effective in improving SP and DP scores of female individuals.

A body of research has been conducted on the effects of Taekwondo training on self-defense. The findings indicate that training in this discipline results in improvements in individuals’ self-defense skills and fosters a sense of security. According to the findings of Yılmaz et al. (2024), a six-week Taekwondo training program yielded favorable outcomes in terms of enhancing self-control, quality of life, and self-defense skills among sedentary women. In their study, Kim et al. (2014) investigated the effect of Taekwondo on the development of basic self-defense skills. The researchers reported that Taekwondo training improved the physical defense skills of the participants and provided improvements in speed, strength, and technique during training. Concurrently, they asserted that the presence of the aforementioned elements contributed to an enhancement in the perceived sense of safety experienced by individuals confronted with peril. Cho et al. (2017) conducted a study that examined how Taekwondo training improves physical and mental self-defense skills in students. The researchers reported that Taekwondo techniques increase physical and mental awareness of self-defense. They also reported that these techniques help participants develop strategic approaches to potentially dangerous situations. In their study, Selucik and Dilek (2020) examined the effects of Taekwondo on self-defense and aggression levels. Their findings indicated a decrease in physical aggression tendencies among individuals practicing Taekwondo and an improvement in self-defense skills. Ghorbanzadeh and Bayar (2013) conducted a study on women that yielded notable findings regarding Taekwondo training. The study revealed that training in Taekwondo imparted two notable benefits to the female subjects. First, the training instilled in them the ability to defend themselves physically. Second, it increased their self-confidence. The female subjects reported that their Taekwondo training had enhanced their capacity to manage perilous circumstances and had fortified their psychological well-being. According to Kim et al. (2021), the practice of Taekwondo has the potential to exert a favorable influence on the psychosocial factors of its participants. Lakes and Hoyt’s (2004) research indicated that Taekwondo enhances not only physical defense skills but also emotional and mental resilience. Individuals who practice Taekwondo develop the ability to maintain composure under stress and to defend themselves effectively. The study also documented enhancements in self-control levels. A substantial body of research has been conducted on the impact of Taekwondo training, and a predominant finding across these studies is that Taekwondo enhances individuals’ self-defense capabilities. This training also fosters the ability to respond to physical attacks in a safe and strategic manner. Furthermore, Taekwondo has been observed to strengthen psychological factors, including mental resilience, self-confidence, and self-control.

In conclusion, six-week regular Taekwondo training had positive effects on quality of life and self-defense skills in healthy female individuals. Although no significant effect on psychological resilience was observed in the short term, it is thought that improvements can be achieved in this area with long-term programs. In this context, Taekwondo training can be considered as a multidimensional intervention strategy that supports women’s physical, mental and social well-being. We believe that these findings will contribute to the existing Taekwondo literature. Taekwondo is widely recognized as a combat sport that provides numerous benefits to its practitioners. However, it is well understood that effective training in this sport requires careful consideration of key parameters such as volume, intensity, duration, frequency and complexity.

The present study is subject to several limitations. The limited sample size of the study resulted in findings that were not widely applicable. The study focused on a six-week time frame and did not evaluate long-term outcomes, which limited the understanding of sustainable effects. The restriction of the study to participants from a single faculty at a single university renders the results applicable only to this specific student population. Physiological and/or biochemical variables were not analysed. The study exclusively included female university students. The study did not address potential gender differences that could influence the results. It is recommended that future research endeavours should include samples of greater size and diversity, whilst also examining long-term effects. Furthermore, it is advised that gender be considered as a variable, in addition to exploring a more extensive range of Taekwondo exercise protocols. This would facilitate the attainment of a more comprehensive understanding. The study’s key strengths are as follows: The study’s design, which entailed the comparison of two physically active groups (TKD) and an inactive group (CG), along with the random assignment of participants at the outset, enhanced the internal consistency of the study. The use of validated assessments commonly used in the scientific literature increased external validity. Finally, the use of training programmes adapted to the characteristics of female university students reduced the risk of injury and increased compliance with interventions.

## 5 Conclusion

This study provides significant evidence that the integration of Taekwondo martial arts exercises into the academic curriculum of female university students results in substantial improvements in various domains of sports psychology and mental health. The extant literature suggests that Taekwondo martial arts exercises in a non-hostile environment at the beginner level enhance psychological resilience, self-control, and quality of life levels. The present study revealed that 6 weeks of Taekwondo training resulted in significant improvements in female participants’ physical health, mental health, defence against dangerous physical attacks (DP) and defence against simple physical attacks (SP). However, no significant difference was observed between the groups in terms of psychological resilience scores. The findings of the present study suggest that Taekwondo may be an effective exercise method for improving women’s quality of life. This finding suggests that Taekwondo exercises have the potential to function as an intervention tool for society, contributing to not only psychological well-being but also social participation and equality.

## Data Availability

The datasets presented in this study can be found in online repositories. The names of the repository/repositories and accession number(s) can be found in the article/supplementary material.

## Footnotes

^1^ https://www.randomizer.org/
